# Changes in smell and taste perception related to COVID-19 infection: a case–control study

**DOI:** 10.1038/s41598-022-11864-8

**Published:** 2022-05-17

**Authors:** Camilla Cattaneo, Ella Pagliarini, Sara Paola Mambrini, Elena Tortorici, Roberto Mené, Camilla Torlasco, Elisa Perger, Gianfranco Parati, Simona Bertoli

**Affiliations:** 1 Sensory & Consumer Science Lab (SCS_Lab), Department of Food, Environmental and Nutritional Sciences (DeFENS), University of Milan, 20133 Milan, Italy; 2 International Center for the Assessment of Nutritional Status (ICANS), Department of Food, Environmental and Nutritional Sciences (DeFENS), University of Milan, 20133 Milan, Italy; 3 Laboratory of Metabolic Research, San Giuseppe Hospital, IRCCS Istituto Auxologico Italiano, 28824 Piancavallo, Italy; 4 Department of Cardiovascular Neural and Metabolic Sciences, San Luca Hospital, IRCCS Istituto Auxologico Italiano, 20149 Milan, Italy; 5 Department of Medicine and Surgery, University of Milano-Bicocca, 20126 Milan, Italy; 6 Sleep Disorders Center and Department of Cardiology, San Luca Hospital, IRCCS Istituto Auxologico Italiano, 20149 Milan, Italy; 7 Obesity Unit - Laboratory of Nutrition and Obesity Research, Department of Endocrine and Metabolic Diseases, IRCCS Istituto Auxologico Italiano, 20145 Milan, Italy

**Keywords:** Physiology, Diseases

## Abstract

The main aim of the present study was to psychophysically evaluate smell and taste functions in hospitalized COVID-19 patients and to compare those results with a group of healthy subjects. Another aim of the study was to assess the relationship of changes in patients’ smell and taste functions with a number of clinical parameters, symptoms, and other physiological signs as well as with severity of disease. Olfactory and gustatory functions were tested in 61 hospitalized patients positive for SARS-CoV-2 infection and in a control group of 54 healthy individuals. Overall, we found a significant impairment of olfactory and gustatory functions in COVID-19 patients compared with the control group. Indeed, about 45% of patients self-reported complaints about or loss of either olfactory or gustatory functions. These results were confirmed by psychophysical testing, which showed a significantly reduced performance in terms of intensity perception and identification ability for both taste and smell functions in COVID-19 patients. Furthermore, gustatory and olfactory impairments tended to be more evident in male patients suffering from more severe respiratory failure (i.e., pneumonia with need of respiratory support need during hospitalization).

## Introduction

The recent ongoing outbreak of coronavirus disease 2019 (COVID-19), the novel coronavirus infective syndrome due to SARS-CoV-2, is still a clinical problem which has caused widespread fatalities on a global scale (WHO coronavirus disease dashboard, https://covid19.who.int).

The most common symptoms observed in COVID-19 patients included fever, cough, diarrhea, rhinorrhea, sore throat and shortness of breath, though some patients developed severe pneumonia, acute respiratory distress syndrome, and multiple organ failure resulting in death^1^. Moreover, in parallel with the pandemic spread to more countries and with the escalation in the numbers of infected individuals, olfactory and gustatory dysfunctions began to be known to everyone, since have been increasingly reported in a great number of patients^2,3^.

Many viruses (e.g., rhinovirus and some coronavirus) may lead to taste and smell dysfunction through a nasal mucosa inflammatory reaction and rhinorrhea development. However, the taste and smell dysfunctions linked to COVID-19 infection seems to be characterized by peculiar features and a number of hypotheses regarding their pathogenetic mechanisms have been raised^4–9^.

In Italy, the prevalence in adult COVID-19 patients of either taste or smell disorders is reported to be around 33.9%, while 18.6% reported both dysfunctions^10^. A bunch of multicenter studies performed in Europe reported that more than 80% of COVID-19 patients presented smell and/or taste dysfunctions^11–13^. However, the reliability of these reports has been criticized because of the subjective nature of collected data, mainly based on questionnaires, self-reports or information from a patient’s electronic health records. Surveys carried out on the basis of subjective symptoms only may indeed have been affected by important biases, thus limiting their ability to estimate the real prevalence of anosmia and ageusia in COVID-19 patients^3,14^.

In order to reduce this bias, a handful of studies have tested the dysfunction with psychophysical tests (e.g.,^15–24^). However, the majority of them mainly used self-administered^17–21^ or short version tests of olfactory and gustatory functions^16,24^, which have been found to be unreliable and inaccurate^25,26^. Moreover, at the time when the present paper was written, few studies included a control group of healthy subjects in their protocol^9,27–29^. Therefore, given the importance of a psychophysical determination of taste and smell impairments for the early diagnosis of COVID-19, our study was aimed to psychophysically evaluate smell and taste functions in hospitalized COVID-19 patients and to compare data from these patients results with those collected in a group of healthy subjects, according to a study design rarely implemented so far. A second aim of our study was to investigate the relationship of data related to smell and taste disorders with a number of clinical parameters in COVID-19 patients and with the overall severity of this disease.

## Results

### Cohort description

Overall, we collected data from 115 subjects of which 61 were hospitalized patients (COVID, cases group) with laboratory-confirmed SARS-CoV-2 infection and 54 healthy individuals (CTRL, controls group). In the cases group, 27 (44.3%) of patients were female and 34 (55.7%) were male, with a mean age of 59.3 ± 10.2 years (range 28–82). In the control group, 27 individuals (50.0%) were female and 27 (50.0%) were male, whilst the mean age was 56.8 ± 12.1 years (range 25–75). No differences in terms of gender (χ^2^ = 0.38, p = 0.54), age distribution (t = -1.20, p = 0.23) or smoking habits (χ^2^ = 2.22, p = 0.33) between the case and control groups were observed. A higher proportion of overweight/obese were found in the cases group, while controls were more likely to be normal weight (χ^2^ = 11.59; p < 0.001).

The general and clinical features of the cases and controls are shown in Table 1.

**Table 1:** General and clinical features of cases and controls.

	Cases (COVID)	Controls (CTRL)
	n = 61	n = 54
Gender (F:M)	27:34	27:27
Age (mean ± SD)	59.3 ± 10.2	56.8 ± 12.1
BMI (mean ± SD)	27.0 ± 6.5	23.6 ± 3.6
**Smoking habits**		
Never smokers, n (%)	30 (49.2%)	32 (59.3%)
Former smokers, n (%)	25 (41.0%)	15 (27.8%)
Current Smokers, n (%)	6 (9.8%)	7 (12.9%)
**Reported symptoms**		
Fever, n (%)	43 (70.5%)	
Cough, n (%)	14 (22.9%)	
Dyspnea, n (%)	21 (34.4%)	
Gastrointestinal symptoms, n (%)	3 (4.9%)	
Myalgia, n (%)	2 (3.3%)	
Olfactory complaint (hyposmia/anosmia) n (%)	28 (45.9%)	
Gustatory complaint (dysgeusia/ageusia) n (%)	27(44.3%)	
Pneumonia at chest imaging, n (%)	39 (63.9%)	
Respiratory support during hospitalization, n (%)	30 (49.2%)	
**Days of symptoms onset**		
< 20 days	*35 (57.4%)*	
> 20 days	*26 (42.6%)*	
**Comorbidities**		
Hypertension, n (%)	20 (32.7%)	
Diabetes, n (%)	8 (13.1%)	
Cardiovascular disease, n (%)	13 (21.3%)	
Overweight/Obesity, n (%)	35 (57.4%)	14 (25.9%)

**Figure 1:**
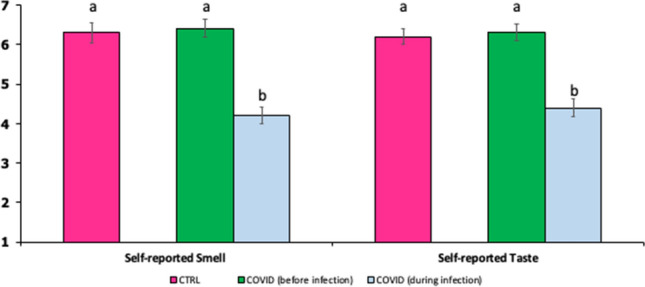
Mean intensity self-reported scores ± SEM of smell and taste functions of controls grouped by controls (CTRL, magenta bars) at the time of enrollment and cases (COVID) before (green bars) and during infection (light blue bars). Different letters indicate significant differences according to Mann–Whitney U test; *P* < 0.001).

About 45% of patients within the COVID group had complaints related to taste and smell, or reported loss of either olfactory or gustatory functions, whilst no individual in the control group complained of such symptoms. Most of the patients stated that they had difficulties to identify odors or reported qualitative changes in olfactory ability (parosmia and phantosmia) and/or stated that foods and beverages were generally perceived as tasteless. Moreover, some patients highlighted that perceived saltiness was particularly increased during their illness. The self-reported scores of smell and taste functions of controls (at the time of enrollment) and cases (before and during infection) were reported in Fig. 1. Significant differences (*P* < 0.001) were highlighted in self-reported smell and taste scores between COVID-19 patients before and during the infection.

Correlations among demographic factors (i.e., age and BMI) and the variables of interest related to smell and taste (Identification Smell Score, Intensity Smell Score, Irritation Smell Score, Identification Taste Score, Intensity Taste Score) in both cases and controls are shown in Table 2. For the control group, BMI was negatively correlated with taste variables and with the Intensity Smell and Irritation Smell score. Moreover, in both groups almost all the variables of interest related to smell and taste were positively (*P* < 0.05) correlated to each other.

**Table 2:** Correlations among demographic factors (i.e., age and BMI) and the variables of interest related to smell and taste (Identification Smell Score, Intensity Smell Score, Irritation Smell Score, Identification Taste Score, Intensity Taste Score) in Control and COVID-19 groups.

	Age	BMI	Identification Smell Score	Intensity Smell Score	Irritation Smell Score	Identification Taste Score	Intensity Taste Score
**Controls**							
Age	1	− 0.015	0.053	− 0.087	− 0.074	0.121	0.094
BMI		1	0.105	− 0.260**	− 0.364**	0.215*	− 0.225*
Identification Smell Score			1	0.249**	0.192	0.280*	0.296**
Intensity Smell Score				1	0.241*	0.203	0.519***
Irritation Smell Score					1	0.188	− 0.152
Identification Taste Score						1	0.149
Intensity Taste Score							1
**Covid-19**							
Age	1	− 0.048	− 0.120	− 0.128	0.046	− 0.195	0.026
BMI		1	0.079	0.161	0.052	− 0.090	− 0.002
Identification Smell Score			1	0.423***	0.089	0.223*	0.263**
Intensity Smell Score				1	0.051	− 0.055	0.457***
Irritation Smell Score					1	0.049	0.285**
Identification Taste Score						1	0.401**
Intensity Taste Score							1

### Effect of COVID-19 infection on olfactory and gustatory functions

**Figure 2:**
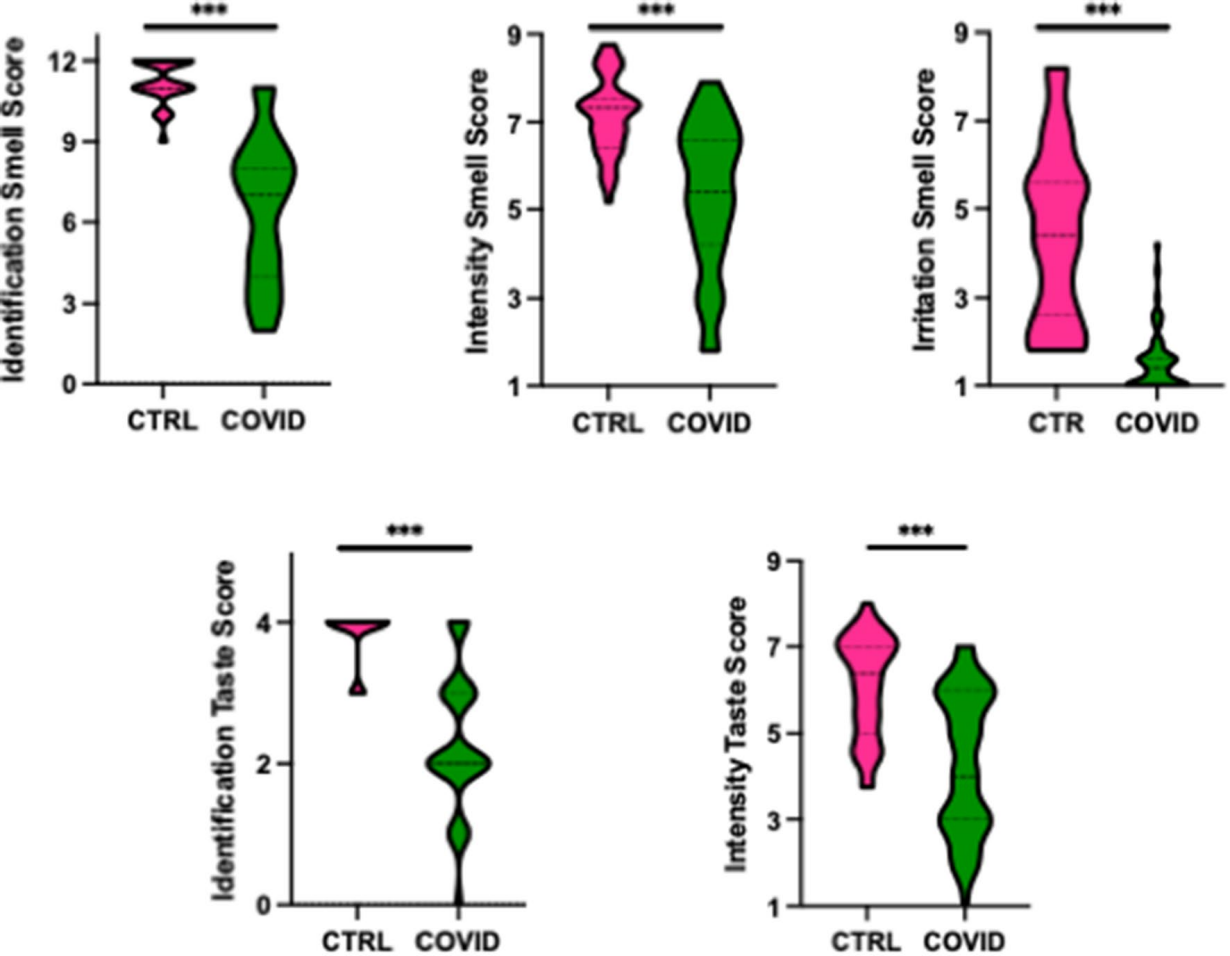
Violin plots showing the differences in olfactory and gustatory assessments in controls (CTRL) and cases (COVID). The plots provide a representation of data distribution and the median ± IQR within each group for each smell and taste assessments. Statistics according to Mann–Whitney U test; ***P < 0.001).

The effect of the presence or absence of infection on the olfactory and gustatory functions in cases and controls was tested applying Mann–Whitney U statistics and the results are shown in Fig. 2. COVID-19 patients significantly (*P* < 0.001) differ from controls in terms of performance in both smell and taste. COVID subjects presented impairments in Identification Smell Score (z = − 8.7), Intensity Smell Score (z = − 6.2), Irritation Smell Score (z = − 8.4), Identification Taste Score (z = − 7.8) and Intensity Taste Score (z = − 5.5). Regarding the subject’s ability to identify a basic taste, sweet and bitter taste were shown to be more impaired with about 60% of the COVID-19 patients unable to recognize these two tastes.

### Comparison of control and case groups on odors’ hedonic response

**Figure 3:**
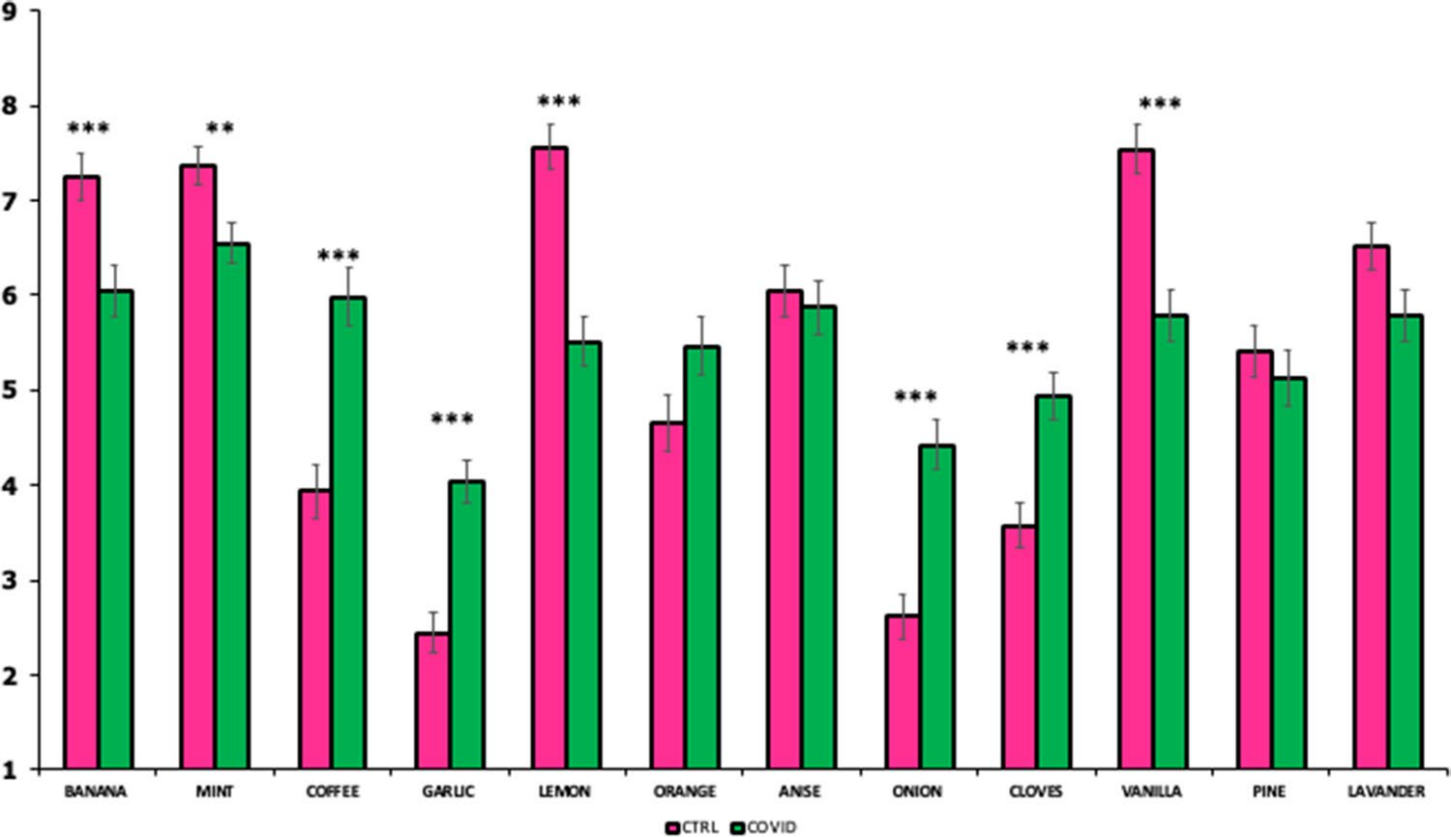
Mean liking ± SEM for the individual odors grouped by controls (CTRL, magenta bars) and cases (COVID, green bars). Statistics according to t- test; **P < 0.01; ***P < 0.001).

An overall difference was seen between groups (COVID *vs* CTRL) for liking, where the controls generally reported higher liking scores compared to the cases. With regard to the individual odors (Fig. 3), controls reported higher liking scores for the most pleasant odors compared to the COVID group (banana: t = 3.3; p < 0.001, mint: t = 3.0; p < 0.01, lemon: t = 6.1; *P*< 0.001 and vanilla: t = 4.9; p < 0.001), whereas the inverse was seen for coffee (t = − 4.6; *P* < 0.001), garlic (t = − 5.4; *P* <  0.001), onion (t = − 5.1; *P* < 0.001) and cloves (t = 3.9; *P* < 0.001), which were more liked by the COVID group.

### COVID-19 patients’ segmentation and clusters’ characterization

**Figure 4:**
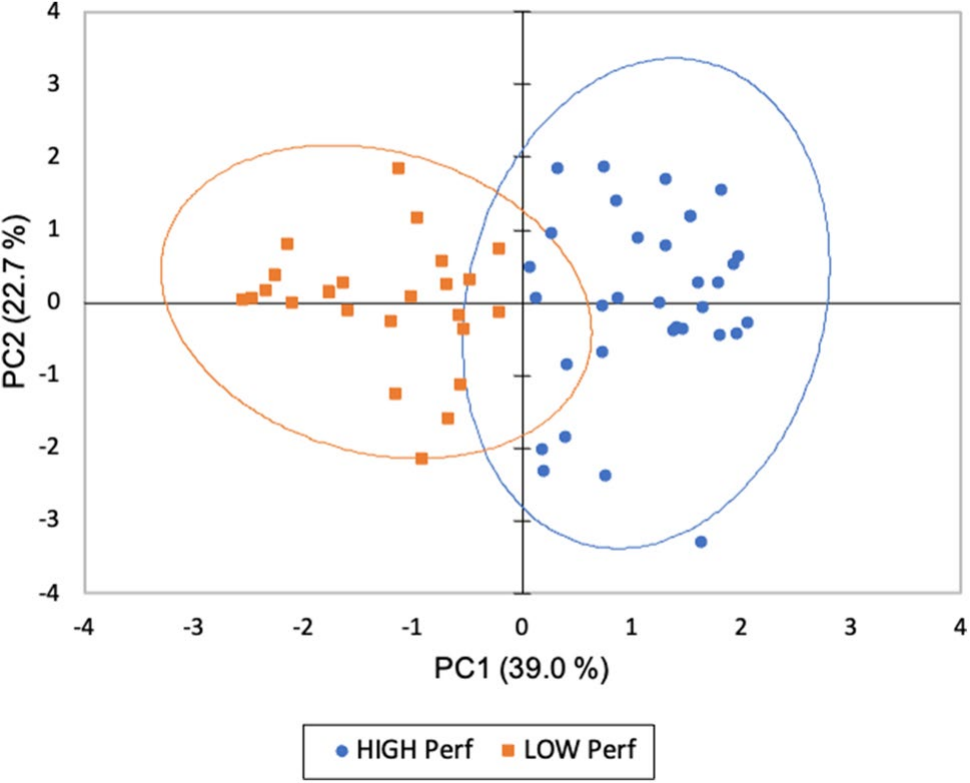
Bi-plots from Principal Component Analysis with confidence ellipses (confidence level 95%) conducted on variables related to smell and taste measurements (Identification Smell Score, Intensity Smell Score, Irritation Smell Score, Identification Taste Score, Intensity Taste Score) obtained for COVID group (n = 61). Patients characterized by a low and high sensory performance are displayed in orange (*LOW Perf*) and blue (*HIGH Perf*), respectively.

The PCA performed on the sensory data provided by each patient resulted in two dimensions accounting for 61.7% of variance in the data (Fig. 4).

Looking at the bi-plot, the first PC (from the left to the right of the plot, PC1) separates the COVID-19 patients according to their olfactory and gustatory performance. Hence, based on loading plot scores, patients were divided in two clusters. One cluster of COVID-19 patients, on the left side of the map, present lower scores for all the variables investigated (Low performance, *LOW Perf*: n = 28; 46%); and one cluster, on the right side of the map, presented higher values for all the scores related to olfactory and gustatory functions (High performance, *HIGH Perf*: n = 33; 54%).

Cluster characterization by age, BMI, gender, smoking habits, stage of disease and severity of symptoms (i.e., pneumonia and respiratory support during hospitalization) is reported in Table 3. According to t-test results, there were no differences according to age and BMI in the two clusters. Moreover, no differences according to smoking habits and stage of disease (i.e., days of symptom onset) distributions were found between *HIGH Perf* and *LOW Perf*. Small differences, although not significant (*P* < 0.10), were found in gender distributions and severity of symptoms. Indeed, *Cluster LOW Perf* is characterized by higher percentages of male patients with severe pulmonary alterations such as a diagnosis of pneumonia and the need of respiratory support during the hospitalization.

**Table 3:** Clusters’ characterization.

	Cluster High Perf	Cluster Low Perf	t	*P*
Age (mean ± SEM)	58.7 ± 1.8	60.0 ± 1.9	− 0.5	0.60
BMI (mean ± SEM)	27.8 ± 1.1	26.2 ± 1.2	0.9	0.33
			**χ**^**2**^	*P*
**Gender (%)**			3.1	0.08
Female	54	32		
Male	46	68		
**Smoking habits (%)**			2.8	0.25
Daily	16	4		
Quit	42	39		
Never	42	57		
**Pneumonia (%)**			2.7	0.09
Yes	54	75		
No	46	25		
**Respiratory support (%)**			2.7	0.09
Yes	39	61		
No	61	39		
**Days of symptom onset (%)**			1.0	0.31
< 20 days	52	64		
> 20 days	48	36		

## Discussion

Our study provided a psychophysical quantification of the odor and taste identification capacity in hospitalized COVID-19 patients by means of a reproducible, easy to use, fast, cheap and disposable system, according to a case–control study design, rarely implemented so far. Moreover, our study provided information on the relation of taste and smell alterations with clinical parameters, symptoms, and other physiological signs as well as with severity of COVID-19.

Our cohort was mainly composed of symptomatic patients with evidence of pneumonia at chest imaging (63.9%), and about half of patients had required some form of respiratory support at some point during disease course. Therefore, this cohort is mostly representative of the moderate-to-severe spectrum of COVID-19. About 45% of patients within the COVID group self-reported complaints regarding smell and taste, or even complete loss of either olfactory or gustatory functions. Indeed, when the patients were asked to subjectively judge their senses of smell and taste before and during the infection using a continuous 7-point scale, a clear drop of about 2 points in overall scores was highlighted, indicating a progressive increase in olfactory and gustatory impairments. These data have been confirmed by the psychophysical quantification performed in our study using a reproducible disposable system, showing a significantly lower performance in terms of intensity perception and identification ability in both taste and smell in COVID-19 patients as compared to controls.

Our findings are in line with previous observations based on subjective reports^12–14,30,31^ or psychophysical evaluations^15–24^, and with the previous studies which involved a control group of healthy subjects^9,27–29^. Moreover, we found that sweet and bitter taste were shown to be more impaired in comparison with the other basic taste. Indeed, about 60% of cases were not able to properly identify such taste, as previously suggested by Huart et al*.*^9^. This is an interesting result since sweet and bitter taste share many common features in the transduction mechanisms (i.e., G-Protein coupled receptors, GPCRs). Individuals affected by these sensory alterations may present changes in eating habits, such as an increased consumption of food rich in sugars and fat to compensate the deficiency^32,33^, which in turn may negatively affect a number of associated clinical conditions such as hypertension, obesity, diabetes and cardiovascular diseases^34^.

Although the pathogenetic mechanisms are not fully understood, the currently available evidence suggests that the most likely cause of anosmia during COVID-19 infection is an altered function of olfactory sensory neurons left vulnerable and deprived of nutrients, due to the infection and the death of supporting cells, microvillar cells, and vascular pericytes which greatly express angiotensin-converting enzyme 2 (ACE2)^5,6^. Concerning taste impairments, Xu and colleagues^35^ demonstrated that epithelial cells of the basal region of filiform papillae on the tongue express ACE-2 receptors at a significant level. Thus, the pathogenesis of taste dysfunctions may comprise indirect taste receptors damage through infection of epithelial cells and subsequent local inflammation^7,36^. Moreover, it should be further investigated whether the expression or function of the sweet and bitter receptors could be modified by SARS-CoV-2 infection and, particularly, GPCR may also be possible targets of the virus^9^.

Focusing the attention only on case group and applying a PCA to identify patients’ segments according to specific sensory function pattern, the results shown that gustatory and olfactory impairments seem to characterize male patients suffering from severe pulmonary alterations (i.e., pneumonia and need of respiratory support during hospitalization).

Although the taste and smell impairments are not as well studied as the loss of other senses such as vision and hearing, researchers know that the consequences can be severe and have a clinical relevance. Indeed, the persistence of smell and taste dysfunctions may have health and social consequences^37,38^. In the present study, olfactory disorders have been evaluated taking into account both quantitative assessment (i.e., perception of odor intensity) and qualitative symptoms (i.e., odor pleasantness/unpleasantness). The hedonic evaluation was performed in order to study the possible impact of smell impairments in liking, since it is an important aspect of food intake and relates to the immediate response to an odor. It has been suggested that the severe decline in perceived intensity is likely to affect odor liking. In this context, the lack in the ability to experience the smell of different odors could compromise the eating experience, which may become bland and confusing. Consequently, this has implications for dietary behavior and nutritional health as food intake may be compromised by the ability to enjoy and adhere to a sufficient and varied diet^37,39^. Unexpectedly, odors that are usually identified as common triggers of parosmia or phantosmia disorders (i.e., coffee, garlic and onion;^40^) were scored as more pleasant by the COVID group than the control group. In most cases of parosmia when patients inhale odor molecules, their perception does not match their previous memories of such odors and they commonly associate this perception with disgust and revulsion^41,42^. Nevertheless, there have been cases in which this mismatch was judged as pleasant^43^. One explanation for this shift to more positive hedonic evaluation may lie in the reduced olfactory and chemosensitive perception of COVID-19 patients. Indeed, some of the key aroma compounds responsible for the strong smell and the stimulation of the trigeminal nerve (i.e., the variety of potent sulfur-compounds of garlic and onion) could not be perceived at all by COVID-19 patients. In the absence of these strong odors, other pleasant compounds may dominate olfactory perception^42,44^. To the best of our knowledge, no previous study has ever compared the hedonic scores obtained in a case–control study involving COVID-19 patients, therefore further data are needed to allow a deeper interpretation of results and a better understanding of their pathogenetic mechanisms.

Although the present research was among a little bunch of studies that psychophysical evaluate smell and taste disorders comparing COVID-19 patients and a group of healthy controls, a few limitations should be acknowledged. Firstly, the use of non-validated method, such as Sniffin’ Sticks kit, to evaluate olfactory performance. The choice to develop and validate a disposable smell kit has been made in order to avoid any type of cross-infection performing the test. Secondly, the olfactory and gustatory testing could not be performed on patients who were in the intensive care unit, which prevented us from investigating the possible relationship of smell and taste impairments with the most severe forms of this disease. On the other hand, we could not investigate the prevalence of smell and taste alterations in asymptomatic/pre-symptomatic patients confined at home on quarantine, which did not allow us to investigate the possible early occurrence of these symptoms and the possibility to use them for a very early diagnosis of SARS-CoV-2 infection. Finally, the relatively small number of study participants, the single clinical center in which the study was performed, and the homogeneous patient population involved (i.e., same age distribution) could somehow limit the generalization of our findings.

In conclusion, the sudden loss of smell and taste could continue to represent a useful and specific diagnostic marker to raise the suspicion of COVID-19 infection. Further studies are needed to shed light on the pathophysiological mechanisms associated with these symptoms, both in cases where changes in smell and/or taste are transient and in those where they are permanent. While luckily the majority of cases up to the time of writing this paper have reported temporary olfactory and gustatory dysfunctions, more detailed and long-term studies are needed to follow-up patients suffering from long-term/permanent loss of smell and taste. The impact of these changes on their experience of everyday life and enjoyment of food products, and ultimately on their eating habits, quality of life and cardiometabolic complications should be deepened. Understanding the extent to which patients’ eating attitudes, behaviors and perceptions continue to change or revert back to normal, could represent a novel challenge for both the healthcare systems and the food industry.

## Materials and methods

### Subjects

The study was performed at Istituto Auxologico Italiano, IRCCS, Department of Cardiovascular Neural and Metabolic Sciences, San Luca Hospital, Milan, Italy, where sixty-one SARS-CoV-2 positive patients were recruited in the COVID-19 wards between March 8th and May 7th, 2021. To be enrolled in the study, patients had to meet the following inclusion criteria: adults over 18 years of age, rhino-pharyngeal swab positive for SARS-CoV-2 infection, patient acceptance for participation in the study. On the contrary, the study exclusion criteria were: uncooperative patients, inability to communicate verbally with the operators due to language barriers, invasive and non-invasive mechanical ventilation, oxygen therapy and inability to tolerate room air for less than 30 min, subjective pre-existing olfactory and taste disturbances, history of head trauma, allergic rhinitis, chronic rhinosinusitis, and psychiatric or neurological disorders. All patients had a microbiological diagnosis of COVID-19 by RT-PCR for SARS-COV-2 on naso-oropharyingeal swab.

Fifty-four healthy subjects were recruited as the control group among the health care workers of the Istituto Auxologico Italiano and the employees of the Faculty of Agriculture and Food Sciences of the University of Milan, applying the same exclusion criteria, with rhino-pharyngeal swab negative for SARS-CoV-2 infection performed at least 48 h before the enrollment and without prior COVID-19 infection. Cases were matched for sex and age with controls, as far as possible.

Prior to enrolment, all participants provided their informed consent to enter the study. Informed consent was obtained in written form for all participants in the control group. Due to the exceptional emergency conditions, being all patients recruited at the time when COVID-19 epidemic was at its peak in Northern Italy, all included individuals in the case group provided informed consent for participation in the study verbally in presence of two experimenter. The study was performed according to the principles established by the Declaration of Helsinki and the protocol was approved by the ethics committee of Istituto Auxologico Italiano (Approval registration number 2020_12_15_06).

### General procedure

Before olfactory and gustatory function assessment, both patients and healthy controls were given general information about the aim and the workflow of the study and underwent an interview to collect socio-demographic data (gender, age), the self-reported weight (kg) and height (m), used to calculate the Body Mass Index (BMI) in kg/m^2^, smoking habits, comorbidity or conditions that could be cause for exclusion. Patients' general information, COVID-19 signs and symptoms, PCR test results, thorax computed tomography findings, length of hospital stay, and clinical history were obtained by manual review of the medical charts by the attending physician or nurse during their shifts and recorded on a dedicated clinical research form. At the time of enrollment, healthy subjects were asked to declare how they judge their sense of smell and taste using a continuous 7-point scale (with 1: no sense of smell/taste, 7: optimal sense of smell/taste), with scores from 2 to 6 indicating progressively decreasing severity of hyposmia/dysgeusia^45,46^. Hospitalized patients were asked to declare whether they suffered/were suffering deficiencies in olfactory/gustatory functions and how they evaluated their sense of smell and taste before and during the virus infection. Psychophysical olfactory and gustatory assessments were carried out by the same operator (C.C.) utilizing stringent safety standards and wearing full personal protective equipment (e.g., N95 mask, face protection, protective overalls, and shoe covers). A separate odor and taste set was used for each patient to prevent the risk of contamination. The results obtained in the patients’ group were then compared with those obtained for the controls.

### Olfactory assessments

#### Selection of odorants, validity and reliability of the test

Sixteen odorants were pre-selected from the ones included in the European Test of Olfactory Capabilities^47^ and in the Italian Olfactory Identification Test (IOIT)^48^.

Odorous solutions were dissolved in propylene glycol or mineral oil (4 ml each) and presented in 10 ml commercially available sterile plastic tube. Odorous solutions pertaining to the development of the test were obtained from domestic flavor and fragrance companies (Flavourart srl, Oleggio, Italy; Laboratorio Nonna Ortica, Verbania, Italy).

To ensure homogeneity of the test only odorants were included which were similar to each other in terms of identifiability and intensity. Both identifiability and intensity were rated on 9-point scale. Thus, pre-selected odorants were roughly matched for odor intensity and tested with a group of 20 healthy volunteers (11 F, 9 M; mean age 22 years, SD = 2.1). As reported by Hummel et al.^49^, only those odorants were selected for the final version of the test that had a rating of identifiability ± 25% of the mean identifiability score (mean = 10.2 SD 0.7) and an intensity rating ± 25% of the mean perceived intensity score (mean = 7.1 SD = 0.9) of the 16 odors. In terms of familiarity and pleasantness of the odorants care was taken to include those which are familiar to Italian culture, with the majority characterized by a pleasant smell. This was done with the aim of making the test more acceptable to subjects or patients.

With these premises, 12 odorants were finally selected: anise, banana, cloves, coffee, garlic, lavender, lemon, mint, onion, orange, pine, vanilla. The odorants selected cover a wide range of fragrance: citrus, fruity non-citrus, sweet, wood-like, minty, floral, spicy. Most of these have only olfactory component and, in some case, there is a minimal trigeminal component (e.g., anise, garlic, mint, onion, pine)^50,51^.

To determine the usefulness and validity of the smell test we evaluated its reliability or stability over time. Thus, twenty-three individuals (13 women, 10 men; mean age = 44.1 years, SD = 3.8) were selected from our subject population for re-administration of the smell test at an interval of four weeks from the time of their initial test to quantitatively determine the test–retest reliability. The scores between the two test administrations were extremely stable (Pearson r = 0.89, *P* < 0.001). Accordingly, the Bland–Altman plot reflects a good congruence between the results from the 2 sessions (see Supplementary Fig. S1).

### Measurements

Olfactory function was measured using a 12-item odor identification task with the addition of a pleasantness, intensity and irritation ratings. The operator took off the cap of each plastic tube and presented it at a distance of around 1–2 cm away from the participant’s nostrils. The participants were asked to identify the odor from a list of four possible descriptive terms for each odor, in a forced choice paradigm. A correct answer was evaluated as 1 point, while an incorrect one as 0 point which resulted in an Identification Smell Score (ranged from 0 to 12), computed by the sum of the correct responses.

Liking, intensity and irritation were measured for each odorant on a 9-point scale (1 = extremely disliked; 9 = extremely liked; 1 = extremely weak, 9 = extremely strong; 1 = not at all irritant, 9 = extremely irritant). This kind of scale was chosen as a good compromise between reliability and easiness of use, as previously reported^52,53^. The odorants were presented in a randomized order and an interval of at least 30 s was ensured between each odorant.

### Gustatory assessment

The sense of taste was evaluated in all participants by means of the ‘taste strips method’. Water solutions including four fundamental taste stimuli (citric acid 4 g/kg for sourness; caffeine 3 g/kg for bitterness; sucrose 200 g/kg for sweetness; sodium chloride 15 g/kg for saltiness) were prepared^54^. Filter paper strips of identical dimensions (Indigo Instruments-Cat#33,814-Ctl; 47 × 6 × 0.3 mm) were dipped into the solutions for about 10 s and air-dried overnight at 20 °C^55^. This procedure was performed only once for all strips before the study began and the strips were stored in small glassine envelopes at 4 °C. Participants were presented with the paper strip and asked to place each strip on the middle of their tongue, close their mouths, and suck it at least 5 s to allow the tastants to be sensed by taste receptors. The participants were asked to identify the taste and to score intensity on a 9-point scale (1 = extremely weak, 9 = extremely strong). The participants rinsed their mouth with distilled water between the application of the paper strips. A correct answer was evaluated as 1 point, while an incorrect one as 0 point which resulted in an Identification Taste Score (ranged from 0 to 4), computed by the sum of the correct responses.

### Statistical analysis

Variables of interest were summarized using means ± SD for normally distributed variables or median ± IQR for not normally distributed variables. Shapiro Wilk’s normality test was used to determine the distribution of each variable.

The χ^2^ test was used to evaluate differences on gender, nutritional status and smoking habits proportions between the two groups. The independent samples t-test was used to determinate whether a between group effect occurs for age and BMI.

For descriptive purposes, associations between demographic factors (i.e., age, BMI) and the variables of interest related to smell and taste (Identification Smell Score, Intensity Smell Score, Irritation Smell Score, Identification Taste Score, Intensity Taste Score) were assessed through Pearson or Spearman rank correlation coefficients in both cases and controls.

The statistical analysis of differences in self-reported and tested olfactory and gustatory functions between population sub-groups (CTRL *vs* COVID), was performed using Mann–Whitney U test.

One-way ANOVA models (fixed factor: population sub-groups) followed by Tukey’s HSD test were separately used to assess whether differences exist in hedonic evaluation of the twelve odors.

In order to identify patients’ segments according to specific sensory function pattern and to obtain further information based on the characteristics of the patients and their stage of disease, a map was obtained by means of a Principal Component Analysis (PCA). Hence, patients’ segmentation was performed based on loading scores considering the first two PCs. Differences in olfactory and gustatory functions were verified by Mann–Whitney U test (*P* < 0.05). χ2 tests were computed to evaluate differences on gender, smoking habits proportions, stage of disease and severity of symptoms (i.e., pneumonia and respiratory support during hospitalization) between the clusters in patients’ group. The independent samples t-test was used to determinate whether a between group effect occurs for age and BMI.

All tests were two-tailed, a *P* < 0.05 was considered as significant. The statistical analysis was performed using SPSS 26.1 (IBM, Armonk, New York) and XLSTAT (version 2019.2.2, Addinsoft, Boston, MA, USA) were used for the data analysis. Graphs were performed using the software program GraphPad Prism 9 (GraphPad Software, Inc., San Diego, CA, USA).

## Supplementary Information

Supplementary Figures.

## Data Availability

The datasets generated during and/or analyzed during the current study are available from the corresponding author on reasonable request.
