# Supplementary material for: Changes in smell and taste perception related to COVID-19 infection: a case–control study
Source: Sci Rep. 2022 May 17;12:8192. doi: 10.1038/s41598-022-11864-8 (PMC9112641; doi:10.1038/s41598-022-11864-8)

**Changes in smell and taste perception related to COVID-19 infection: a case-control study**

Camilla Cattaneo^1^, Ella Pagliarini^1*^, Sara Paola Mambrini^2,3^, Elena Tortorici^4^, Roberto Mené^5^, Camilla Torlasco^4^, Elisa Perger^5,6^, Gianfranco Parati^5,6^, Simona Bertoli^2,7^

*^1^Sensory & Consumer Science Lab (SCS_Lab), Department of Food, Environmental and Nutritional Sciences (DeFENS), University of Milan, 20133, Milan, Italy*

*^2^International Center for the Assessment of Nutritional Status (ICANS), Department of Food, Environmental and Nutritional Sciences (DeFENS), University of Milan, 20133, Milan, Italy*

*^3^IRCCS Istituto Auxologico Italiano, Laboratory of Metabolic Research, S. Giuseppe Hospital, 28824, Piancavallo, Italy*

*^4^IRCCS Istituto Auxologico Italiano, Department of Cardiovascular Neural and Metabolic Sciences, San Luca Hospital, 20149, Milan, Italy*

*^5^Department of Medicine and Surgery, University of Milano-Bicocca, Milan, 20126, Italy*

*^6^IRCCS Istituto Auxologico Italiano, Sleep Medicine Center, Department of Cardiology, San Luca Hospital, 20149, Milan, Italy*

*^7^IRCCS Istituto Auxologico Italiano Obesity Unit - Laboratory of Nutrition and Obesity Research, Department of Endocrine and Metabolic Diseases, 20145, Milan, Italy*

*Correspondence to be sent to:* Ella Pagliarini, Sensory & Consumer Science Lab (SCS_Lab), Department of Food, Environmental and Nutritional Sciences (DeFENS), University of Milan, 20133 Milan, Italy

e-mail: ella.pagliarini@unimi.it

Telephone: +39 0250319191

**Supplementary material**

**Fig. S1.** Results from sessions 1 and 2 (test re-test) plotted according to Bland–Altman plot (*x* axis: mean of results from sessions 1 and 2; *y* axis: differences between results from sessions 1 and 2). The coefficient of correlation between results from the 2 sessions was *r* = 0.89 (*p* < .001).


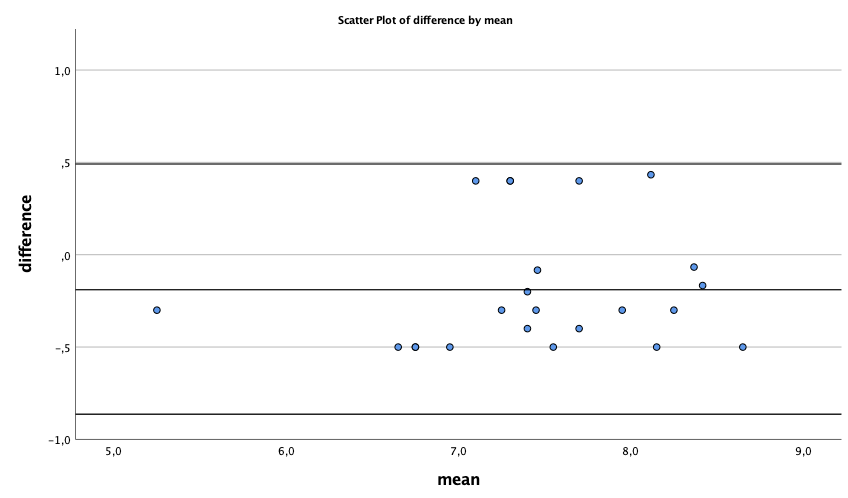

Supplement: Supplementary file 1 — Supplementary Figures. [file 41598_2022_11864_MOESM1_ESM.docx]
